# The paramount of three-dimensional echocardiography in percutaneous closure of large oval perimembranous ventricular septal defect: a case report

**DOI:** 10.1093/ehjcr/ytae170

**Published:** 2024-04-09

**Authors:** Sisca Natalia Siagian, Yovi Kurniawati

**Affiliations:** Division of Pediatric Cardiology and Congenital Heart Disease, Department of Cardiology and Vascular Medicine, National Cardiovascular Centre Harapan Kita, Universitas Indonesia, 11420 Jakarta, Indonesia; Division of Pediatric Cardiology and Congenital Heart Disease, Department of Cardiology and Vascular Medicine, National Cardiovascular Centre Harapan Kita, Universitas Indonesia, 11420 Jakarta, Indonesia

**Keywords:** Echocardiography, 3D TEE, Percutaneous closure, Large oval VSD, Pulmonary hypertension, Heart failure, Case report

## Abstract

**Background:**

Ventricular septal defect (VSD) is the most common type of congenital heart abnormality with perimembranous VSD (pmVSD) accounting for ∼70% of all VSD. Nowadays, transcatheter closure is the first choice for suitable pmVSD. However, there was no report about closing the large oval-shaped VSD percutaneously.

**Case summary:**

A 34-year-old male with known VSD was referred for transcatheter closure after failed attempts in other hospital. Patient had been diagnosed with VSD at a young age, but he was lost to follow-up. He presented with shortness of breath due to heart failure and pulmonary hypertension. The initial measurement of the defect was 6–7 mm by transthoracic echocardiography (TTE), transoesophageal echocardiography (TEE), and LV angiography. However, re-measurement using TEE and 3D echocardiography revealed that the VSD is oval with diameters of 18 mm × 6 mm. Initially, device No. 12/14 was used, but it was dislodged on two attempts. The operator then decided to upsize the device size to No. 16/18 that was successful. The patient’s condition was good, and 6 months follow-up after the procedure showed good outcomes without any residual defect or arrhythmia.

**Discussion:**

In this study, we would like to highlight the rarity of large oval pmVSD that almost failed to be closed with the conventional measurement with echocardiography and fluoroscopy. Transoesophageal echocardiography especially 3D can be the new modality of choice that might be superior to fluoroscopy to decide the right device size in some cases such as oval-shaped pmVSD.

**Learning points**

- We should be aware of the probability of oval shape or ‘not round’ shape of ventricular septal defect (VSD) if the device still dislodged with previous measurement.
- Re-evaluation is a must, and transoesophageal echocardiography examination may be superior to fluoroscopy in some cases in measuring the size and the shape of the defect, such as oval shape.
- Transcatheter closure is the preferred procedure for VSD with decreased EF.
- We have to consider the risk of arrhythmia (atrioventricular block or bundle branch block) when using large devices in large VSD.

## Introduction

Ventricular septal defect (VSD) stands as the predominant congenital cardiac anomaly, with perimembranous VSD comprising ∼70% of all VSD cases.^1^ Treatment modalities encompass surgical repair and percutaneous device closure.^2^ Notably, advancements in interventional techniques within cardiac catheterization have progressed significantly in recent years, rendering percutaneous closure the preferred option. Nevertheless, adherence to specific criteria and careful selection of the occluder’s appropriate size remain crucial considerations.^1,3,4^

## Summary figure

Timeline of the case presentation

**Figure:**
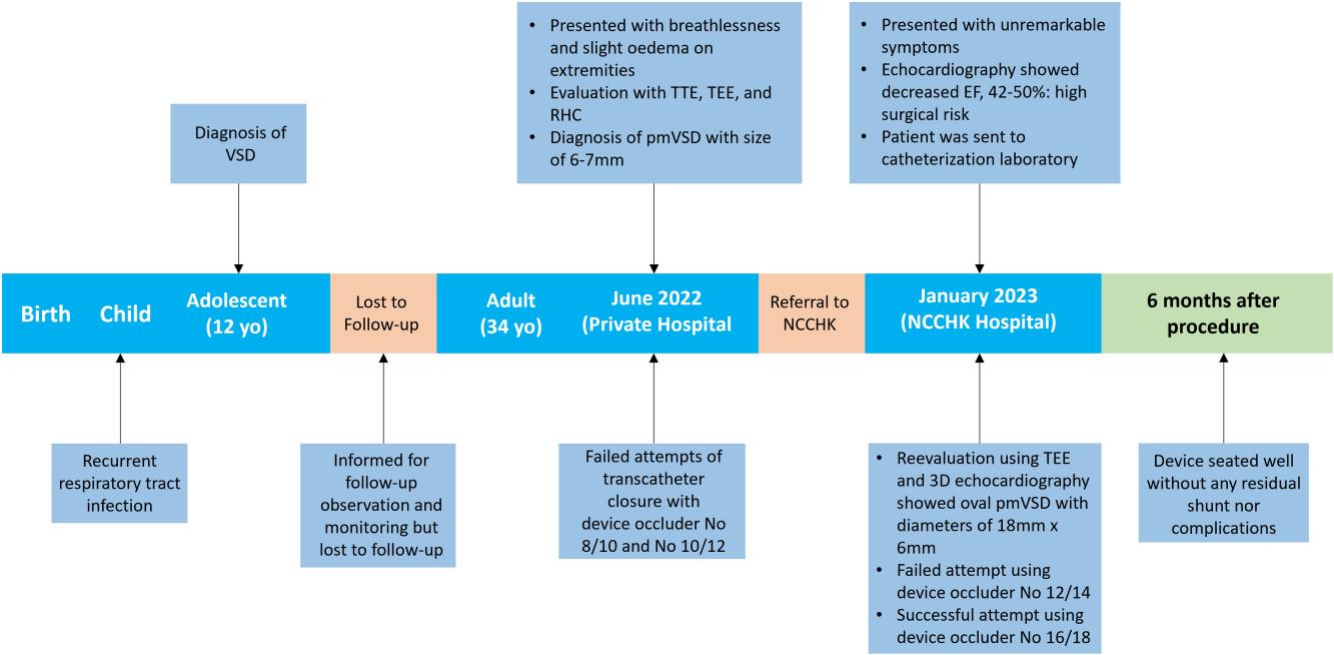


## Case presentation

**Figure 1:**
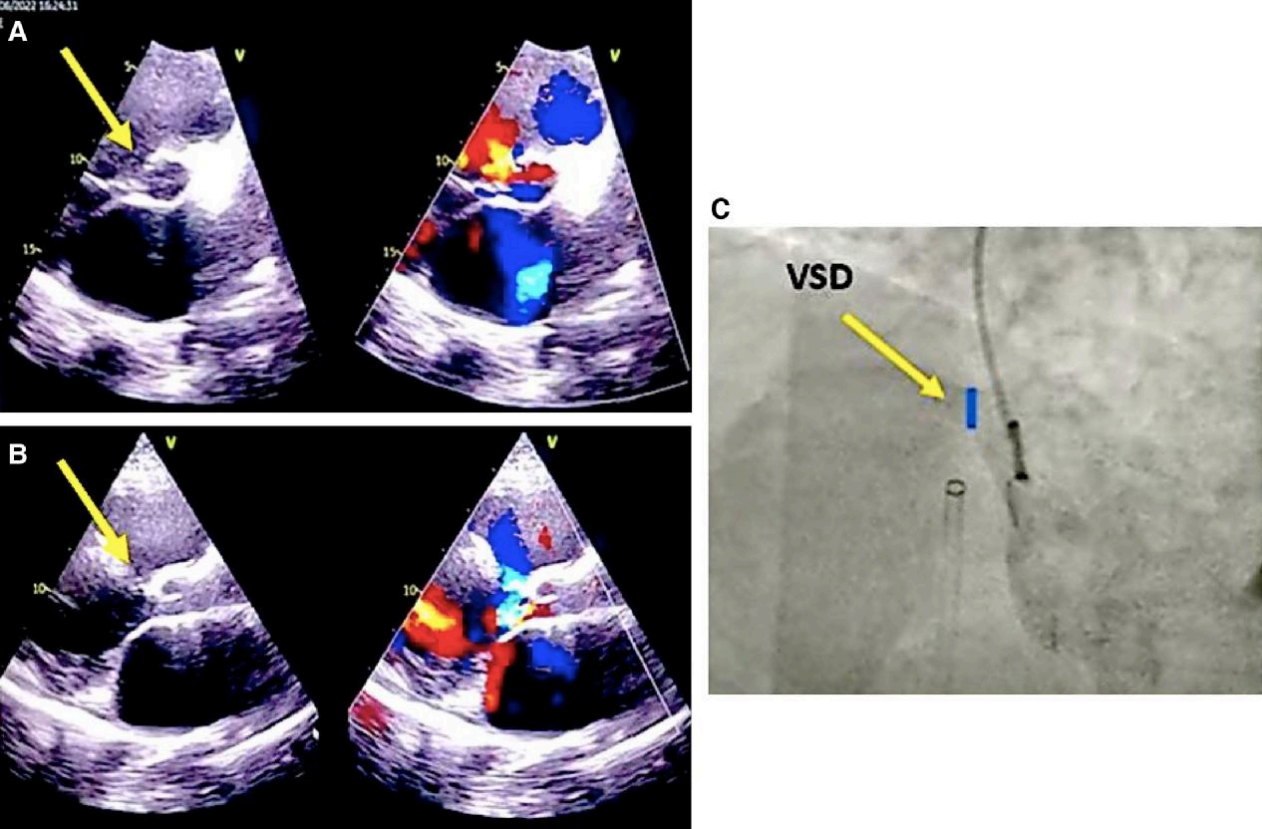
VSD assessment in the previous hospital, defect measurement from TEE (A and B) and LV angiography (C), pointed by the yellow arrows.

A 34-year-old male presented to our centre, reporting shortness of breath a year prior to his current admission, accompanied by a history of unsuccessful intervention at a previous hospital. During childhood, he experienced recurrent respiratory tract infections and was diagnosed with VSD at the age of 12. Unfortunately, he was lost to follow-up and did not receive appropriate treatment thereafter. One year ago, in June 2022, the patient sought medical attention at a private hospital due to heart failure symptoms, including breathlessness and oedema in extremities. Echocardiography and right heart catheterization confirmed perimembranous VSD with pulmonary hypertension, evidenced by a flow ratio of 2.14, pulmonary vascular resistance index 3.6 WU·m^2^, and resistance ratio 0.12. The defect size, measured via echocardiography and left ventricular angiography, was 6–7 mm (*Figure 1*). Despite medication, an attempt at VSD transcatheter closure proved unsuccessful, prompting consideration of surgical correction or a repeat percutaneous procedure at a tertiary centre with enhanced resources.

During the examination at our centre, the patient was compos mentis, with blood pressure of 134/70 mmHg, a regular heart rate of 79 beats per minute, respiratory rate of 30 breaths per minute, oxygen saturation levels of 97%, a temperature of 36.1°C, and weight/height of 94 kg/165 cm. Cardiac examination revealed normal first and second heart sounds, along with a pansystolic murmur, while lungs displayed no rales or wheezing. Other physical examinations yielded normal results. Laboratory tests were mostly within normal limits, except for slightly elevated ALT and AST. ECG indicated sinus rhythm with a heart rate of 75 beats per minute and biventricular hypertrophy. Chest X-ray illustrated cardiomegaly with left ventricular hypertrophy. Echocardiography detailed a large VSD (6–7 mm) with a transVSD pressure gradient of 37 mmHg, mild tricuspid regurgitation (transvalvular gradient of 36 mmHg), moderate mitral regurgitation, and dilated left atrium and ventricle. Left ventricular systolic function was diminished, with an ejection fraction ranging from 42–50% (Teich and eyeballing) and 46% (Simpson), while the left ventricle dimensions at end-diastolic and end-systolic were 92 and 72 mm, respectively.

**Figure 2:**
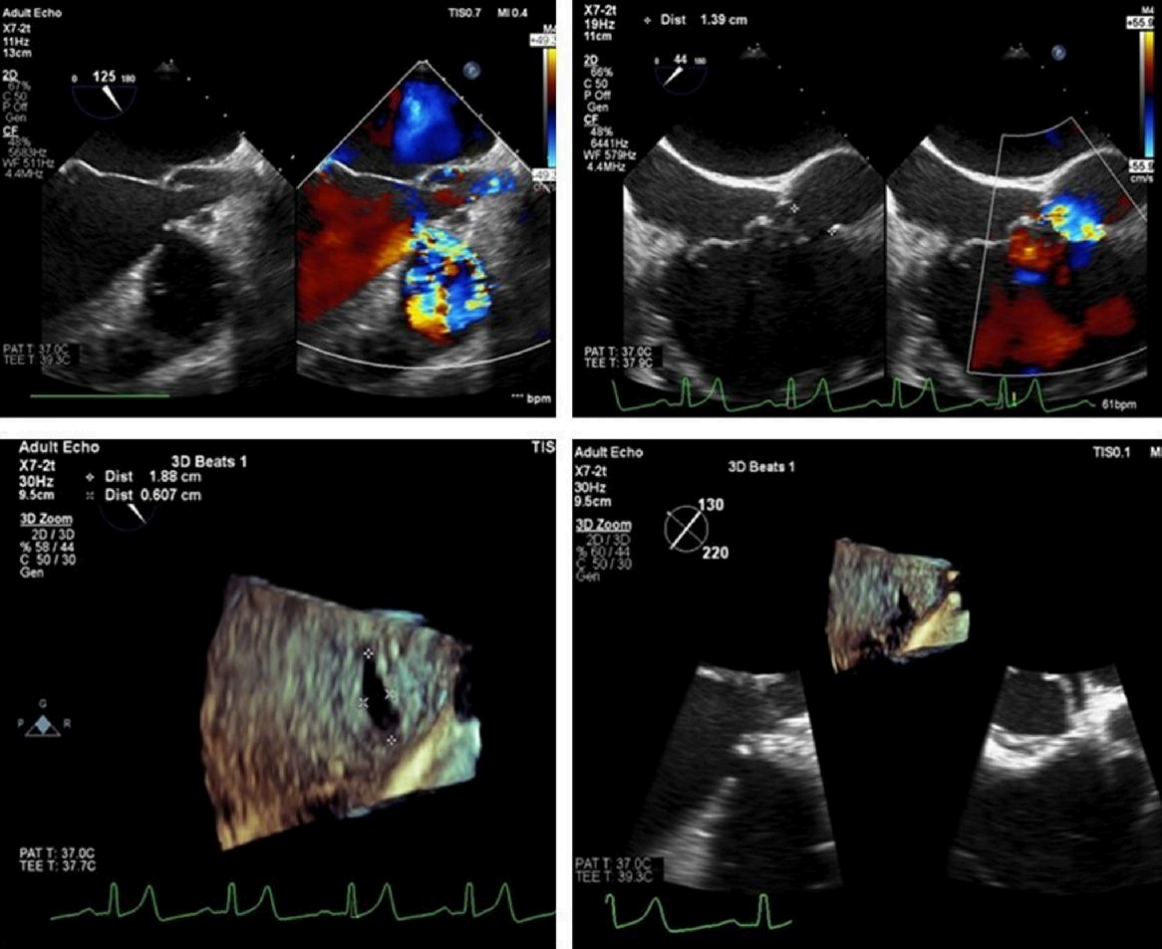
3D echocardiography showing oval-shaped pmVSD with diameter of 18 × 6 mm.

**Figure 3:**
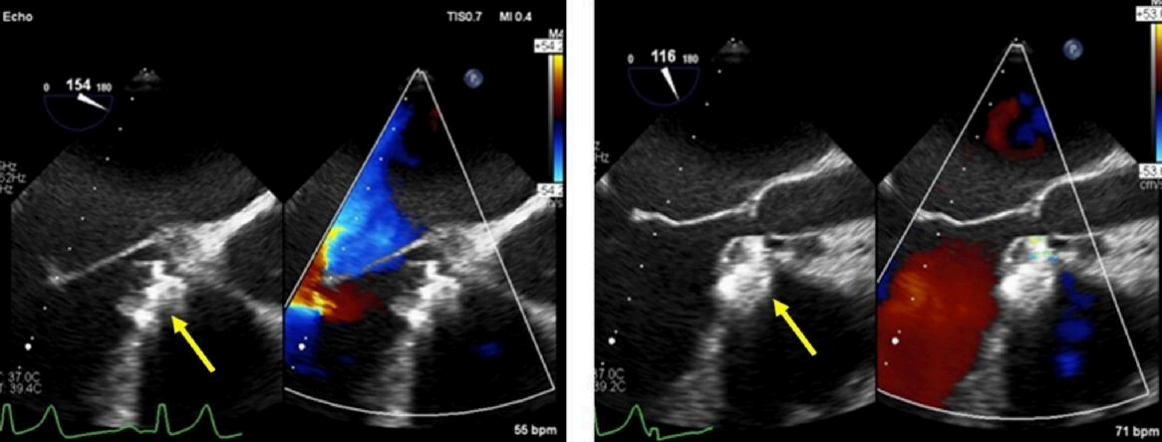
Post-procedural TEE. Device Lifetech 16/18 seated well with no residual of VSD right after the intervention and also during follow-up 1 week and 6 months afterward.

Considering the heightened surgical risk due to decreased ejection fraction and pulmonary hypertension, the patient was deliberated for transcatheter VSD closure with a larger device after consultation with our surgical team. Three-dimensional transoesophageal echocardiography (3D TEE) revealed an oval-shaped VSD with diameters of 18 × 6 mm (*Figure 2*). Despite using the Lifetech HeartR occluder No. 12/14 based on the newly measured size and previous closure attempts, the device proved unstable with a significant peripheral residual shunt, persisting even after two repositioning attempts. To address this, we opted to upsize the device to Lifetech HeartR occluder No. 16/18, acknowledging the potential risk of atrioventricular (AV) block due to AV node disturbance. The delivery sheath was carefully inserted, followed by the device, with TEE guiding each step of the procedure. Deployment on one side was followed by the other. Post-procedural assessment indicated the device stowed in place with satisfactory ECG and haemodynamics, leading to the decision to release the device. Over the subsequent 6 months, the patient exhibited a well-seated device with no residual shunt, valve impingement, or AV block (*Figure 3*).

## Discussion

The natural progression of VSD is contingent upon factors such as its size, location, and the relative resistances in the pulmonary and systemic vascular beds.^1^ Perimembranous defects may, in some cases, spontaneously decrease in size or close through an aneurysm.^2^ In the case presented, the patient was lost to follow-up, allowing the large defect to persist into adulthood. Upon returning to the hospital after decades with dyspnoea, further evaluation revealed heart failure with reduced ejection fraction and pulmonary hypertension. Given the increased risk associated with surgical closure, the balance leaned towards transcatheter closure, supported by advancements in cardiac imaging modalities and techniques, along with diverse occlusion systems. The transcatheter closure of perimembranous VSDs has gained acceptance, although it remains a challenging procedure.^3,4^

Measurements of defect size and distance from the aortic and tricuspid valve are typically confirmed using both angiography and echocardiography.^5^ Most centres use angiography to confirm the size, location, and shunt magnitude, which is the most accurate modality for intraoperative guidance.^6^ Of all the variables considered for device selection, the location, morphology, as well as length and thickness of the edges of the defect are the most important ones.^7^ In our centre, both angiography and TEE were used. Notably, TEE emerged as superior in measuring the defect size, as evidenced by the unexpected discovery of an oval-shaped VSD with diameters of 18 × 6 mm using 3D TEE.

Generally, the selected device is 1–2 mm larger than the maximal diameter of the defect as assessed by TEE and angiography.^7^ Initial attempts with an occluder No. 12/14 were unsuccessful, leading to device dislodgment. A subsequent attempt with the same device resulted in an unstable position with a significant residual peripheral shunt. Re-evaluation of the defect size using 3D TEE prompted the use of a larger device occluder No. 16/18. However, this choice introduced the potential risk of manipulating or causing mechanical trauma to the AV node, possibly inducing AV block or bundle branch block.^8^ After careful consideration of risks and benefits, we proceeded with the procedure, preparing for a transcutaneous pacemaker. The intervention proved successful, with a stable device position, no residual shunt, or complications. A follow-up six months later revealed the patient without complaints and good outcomes, without any delayed complication.

This case underscores the significance of a good and accurate evaluation of a defect before and during a procedure with echocardiography, particularly 3D TEE. Transoesophageal echocardiography holds the most important role in the success of this simple yet complex procedure of transcatheter closure of large oval-shaped perimembranous VSD. While 3D TEE has been widely used in assisting transcatheter ASD closure, its role in percutaneous VSD closure has yet to be fully explored. Balloon sizing is hardly ever used since the interventricular septum is regarded to be a non-stretchable structure.^9^ This case also showed the lack of knowledge on the closure of an oval-shaped VSD. Studies or reports on oval-shaped VSDs are limited, and there is no specific recommendation or guideline on the closure of this type of defect. Therefore, further studies are required for the establishment of recommendations on transcatheter oval shape VSD closure as well as the device selection for this specific defect.

## Conclusion

This study brings attention to the rarity of large oval VSD that posed a significant challenge for closure using conventional measurements with echocardiography and fluoroscopy. The successful outcome was achieved through a comprehensive evaluation of the size and shape of the oval VSD using 3D TEE. This highlights the potential superiority of 3D TEE over fluoroscopy in determining the appropriate device size, particularly in unconventional cases such as oval-shaped VSDs. The findings underscore the evolving role of advanced imaging modalities in enhancing the precision and success of transcatheter closure procedures for complex cardiac defects.

## Supplementary Material

ytae170_Supplementary_Data

## Data Availability

The data underlying this article are available in the article and its online Supplementary material.

## Lead author biography

Sisca Natalia Siagian is a paediatric cardiologist in National Cardiovascular Centre Harapan Kita Hospital, Jakarta, Indonesia. She earned her medical degree, cardiology specialization, and paediatric cardiology subspecialty from Universitas Indonesia in the years of 2006, 2015, and 2018, respectively. She has a keen interest on congenital heart disease from foetal to geriatric, in diagnosing, treatment, and long-term management, and her special objective is in intervention of critical and non-critical congenital heart disease in Indonesia.

## Notes

**Consent:** The patient described in this case signed a standard institutional consent form in accordance with COPE guidelines. The consent contains permission to publish the case for scientific purposes. Every effort has been made to anonymize the data being presented.

**Funding:** None declared.
