# Supplementary figures and images for: The paramount of three-dimensional echocardiography in percutaneous closure of large oval perimembranous ventricular septal defect: a case report
Source: Eur Heart J Case Rep. 2024 Apr 9;8(4):ytae170. doi: 10.1093/ehjcr/ytae170 (PMC11037106; doi:10.1093/ehjcr/ytae170)

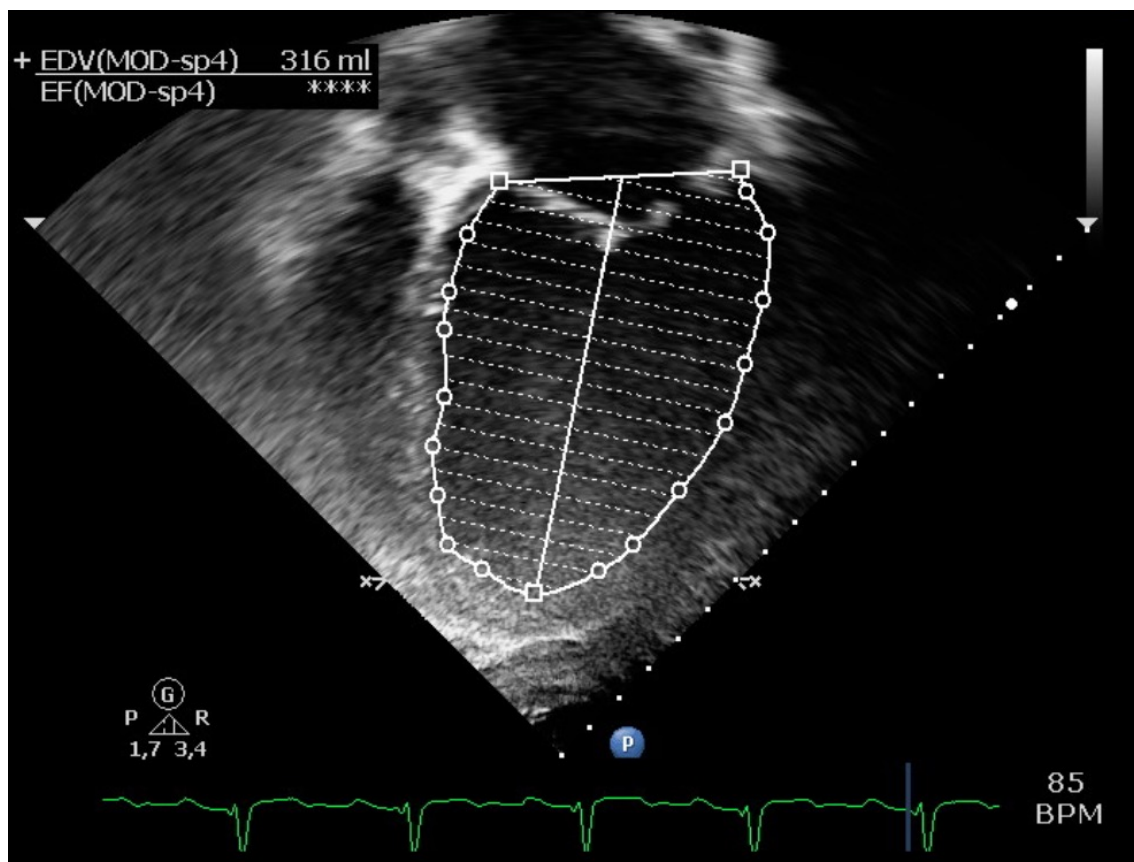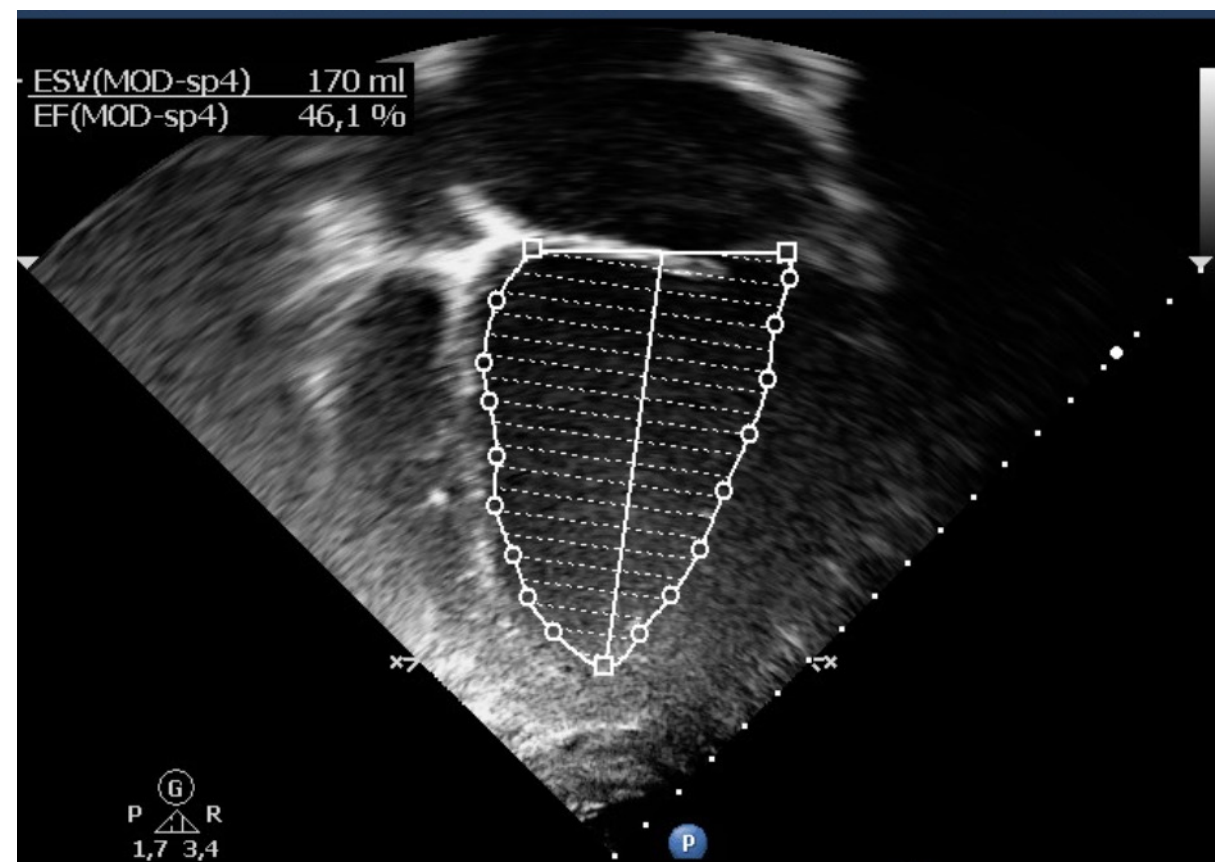

Supplement: ytae170_Supplementary_Data [file ytae170_supplementary_data.zip › Simpson EF.pdf]
